# Hydrogel-Based Innovations in Carpal Tunnel Syndrome: Bridging Pathophysiological Complexities and Translational Therapeutic Gaps

**DOI:** 10.3390/gels11010052

**Published:** 2025-01-09

**Authors:** Venera Cristina Dinescu, Liviu Martin, Marius Bica, Ramona Constantina Vasile, Andrei Gresita, Marius Bunescu, Mihai Andrei Ruscu, Madalina Aldea, Alexandra Daniela Rotaru-Zavaleanu

**Affiliations:** 1 Department of Health Promotion and Occupational Medicine, University of Medicine and Pharmacy of Craiova, 2 Petru Rares Str., 200349 Craiova, Romania; venera.dinescu@umfcv.ro; 2 Faculty of Medical Care, Titu Maiorescu University, Văcărești Road, no 187, 040051 Bucharest, Romania; liviu.martin@prof.utm.ro; 3 Department of Surgery, University of Medicine and Pharmacy of Craiova, 2 Petru Rares Str., 200349 Craiova, Romania; marius.bica@umfcv.ro; 4 Department of Epidemiology, University of Medicine and Pharmacy of Craiova, 2 Petru Rares Str., 200349 Craiova, Romania; ruscumihai@gmail.com (M.A.R.); alexandra.rotaru@umfcv.ro (A.D.R.-Z.); 5 Department of Physiology, University of Medicine and Pharmacy of Craiova, 2 Petru Rares Str., 200349 Craiova, Romania; 6 Department of Occupational Medicine, University of Medicine and Pharmacy of Craiova, 2 Petru Rares Str., 200349 Craiova, Romania; marius.bunescu@umfcv.ro; 7 Department of Psychiatry, University of Medicine and Pharmacy of Craiova, 2 Petru Rares Str., 200349 Craiova, Romania; madalina.aldea@umfcv.ro

**Keywords:** carpal tunnel syndrome, biomaterials, hydrogels, functional hydrogels, peripheral nerve injury, CTS therapy

## Abstract

Carpal Tunnel Syndrome (CTS) is a prevalent neuropathic disorder caused by chronic compression of the median nerve, leading to sensory and motor impairments. Conventional treatments, such as corticosteroid injections, wrist splinting, and surgical decompression, often fail to provide adequate outcomes for chronic or recurrent cases, emphasizing the need for innovative therapies. Hydrogels, highly biocompatible three-dimensional biomaterials with customizable properties, hold significant potential for CTS management. Their ability to mimic the extracellular matrix facilitates localized drug delivery, anti-adhesion barrier formation, and tissue regeneration. Advances in hydrogel engineering have introduced stimuli-responsive systems tailored to the biomechanical environment of the carpal tunnel, enabling sustained therapeutic release and improved tissue integration. Despite these promising developments, hydrogel applications for CTS remain underexplored. Key challenges include the absence of CTS-specific preclinical models and the need for rigorous clinical validation. Addressing these gaps could unlock the full potential of hydrogel-based interventions, which offer minimally invasive, customizable solutions that could improve long-term outcomes and reduce recurrence rates. This review highlights hydrogels as a transformative approach to CTS therapy, advocating for continued research to address translational barriers. These innovations have the potential to redefine the treatment landscape, significantly enhancing patient care and quality of life.

## 1. Background

Carpal Tunnel Syndrome is a prevalent neuropathic disorder caused by compression of the median nerve within the carpal tunnel of the wrist [1]. This neurovascular entrapment manifests symptoms such as dysesthesia, paresthesia, and muscle weakness in the hand and fingers, leading to significant impairment in fine motor skills and sensory functions [2]. CTS primarily affects individuals in occupations requiring repetitive wrist and finger movements, such as data entry, manual labor, and other activities involving repetitive strain, posing a considerable global public health burden [3]. Epidemiological studies estimate a prevalence of approximately 4–5% among adults, with higher incidence rates observed in women and individuals over the age of 40 [2]. Beyond individual disability, CTS imposes substantial healthcare costs and productivity losses, highlighting the urgent need for effective and accessible treatment options [3].

Although both conservative and surgical treatments are available, current interventions often fall short, especially in managing chronic or recurrent CTS [4]. Conservative therapies, including wrist splinting, corticosteroid injections, and physical therapy, frequently provide only temporary relief and fail to address the underlying pathophysiology of nerve compression [5]. CTR surgery, while offering a more definitive solution, is associated with risks such as infection, scarring, and variable recovery outcomes [6]. Post-surgical complications, including perineural fibrosis, often result in recurrent symptoms, necessitating additional interventions [7]. Prolonged recovery times further delay the resumption of daily and occupational activities, underscoring the need for innovative, minimally invasive alternatives capable of delivering sustained therapeutic benefits [8].

Hydrogels, three-dimensional hydrophilic polymer networks, offer unique properties that make them highly suitable for biomedical applications such as drug delivery and regenerative medicine [9]. Their high water content, biocompatibility, and structural resemblance to soft tissues enable localized therapeutic delivery while minimizing systemic side effects [10]. Additionally, hydrogels can be engineered for controlled release of bioactive compounds, providing sustained therapeutic effects tailored to the pathophysiology of CTS [11]. Their injectable, minimally invasive nature and tunable degradation rates position hydrogels as a promising solution for addressing nerve inflammation, compression, and regeneration, particularly in cases where conventional treatments have proven inadequate [12].

**Figure 1:**
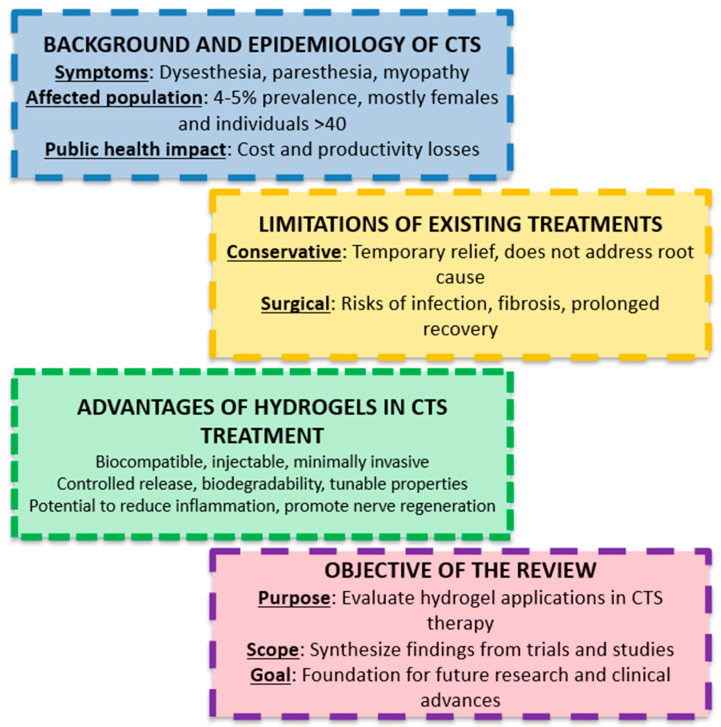
Key insights into Carpal Tunnel Syndrome and the therapeutic potential of hydrogels.

Despite their potential, studies directly investigating the application of hydrogels in CTS are notably lacking, particularly in animal models that replicate compressive nerve entrapment. This research gap limits our understanding of how hydrogel-based therapies could address the specific pathophysiological hallmarks of CTS. This narrative review critically examines existing studies on hydrogel applications in peripheral nerve models that parallel the pathophysiology of CTS. By synthesizing insights from related research and proposing future directions, we aim to underscore the therapeutic potential of hydrogels for advancing CTS treatment (Figure 1). We hope this study stimulates further research into this promising field, encouraging the development of innovative hydrogel-based strategies that could revolutionize the management of CTS and other peripheral nerve disorders.

## 2. Hydrogels: Properties and Applications

Hydrogels are advanced biomaterials composed of polymeric networks capable of absorbing and retaining substantial amounts of water, often several hundred times their dry mass [13]. This exceptional water retention capacity, coupled with their hydrophilic polymer chains, enables hydrogels to form gel-like structures that closely replicate the elasticity and mechanical properties of biological tissues. These attributes render hydrogels highly compatible with in vivo environments [14].

Hydrogels can be synthesized from a diverse range of sources, including synthetic polymers such as polyacrylamide (PAA), polyvinyl alcohol (PVA), and polyethylene glycol (PEG), as well as natural biopolymers like alginate, chitosan, and collagen [15]. Each type of hydrogel offers distinct advantages: synthetic polymers provide precise control over mechanical properties and degradation rates, while natural hydrogels intrinsically promote cellular interactions, often enhancing biocompatibility (Table 1) [16].

A defining characteristic of hydrogels is their exceptional biocompatibility, which stems primarily from their ability to remain hydrated, thereby minimizing immunogenicity and toxicity in biological systems [27]. This hydrated state not only reduces the likelihood of protein adsorption and inflammation but also promotes cellular adhesion and proliferation, facilitating their application in sensitive biomedical contexts [28]. The tunable properties of hydrogels—including porosity, mechanical strength, and degradation kinetics—enable their customization for a wide range of medical applications, including drug delivery, tissue engineering, and regenerative medicine [29]. By modifying polymer crosslinking density or degradation rates, hydrogels can be engineered to deliver bioactive agents, such as anti-inflammatory drugs, growth factors, and neurotrophic proteins, in a controlled and sustained manner. This precision optimizes therapeutic efficacy while minimizing systemic exposure and associated side effects, making hydrogels an ideal platform for targeted and localized therapies [30].

Hydrogels’ adaptability also extends to their surface conformability, allowing seamless integration with soft tissues and complex anatomical structures [31]. This feature enhances their utility in diverse applications, including wound care, ophthalmology, bone repair, and neural regeneration, where they can deliver localized treatments with minimal invasiveness and support tissue regeneration [23,32,33,34]. Collectively, these attributes position hydrogels as a versatile and promising tool across various medical disciplines, meeting the unique demands of different therapeutic contexts.

### Physical and Chemical Properties of Hydrogels for CTS-Specific Applications

In the context of CTS, hydrogels present a particularly promising therapeutic approach by enabling the localized delivery of anti-inflammatory agents or neuroregenerative compounds directly to the carpal tunnel [35]. This targeted delivery system provides a sustained therapeutic effect, potentially addressing the limitations of conventional systemic treatments, which often fail to resolve the underlying mechanisms of nerve compression. By closely mimicking the mechanical properties of soft tissues and facilitating controlled, site-specific drug release, hydrogels offer an innovative pathway for CTS management. This approach has the potential to alleviate symptoms, enhance functional recovery, and improve clinical outcomes, representing a significant advancement in the treatment of this debilitating condition [30].

From a physical standpoint, hydrogels designed for CTS must exhibit an elastic modulus comparable to soft tissue, typically ranging from 0.1 to 10 kPa. This ensures they can alleviate pressure on the median nerve while maintaining mechanical integrity under repetitive motion [25]. The compressive and tensile properties of hydrogels can be finely tuned by adjusting crosslink density and polymer concentration [36]. In CTS applications, hydrogels must endure cyclic compressive stress without fracturing, maintaining stability in the dynamic wrist environment [37]. High water content, often exceeding 90% by weight, further enhances their ability to mimic the extracellular matrix and support cell interaction [38]. The swelling ratio, governed by polymer hydrophilicity and crosslink density, must strike a balance—avoiding excessive swelling that could exacerbate nerve compression while preserving mechanical integrity [30].

Injectability and conformability are critical features for CTS-targeted hydrogels. Injectable hydrogels must exhibit low viscosity during injection and rapid gelation upon placement for minimally invasive delivery [39]. Shear-thinning hydrogels, which temporarily reduce viscosity under applied force and recover their structure post-injection, are particularly advantageous, as they conform to the confined geometry of the carpal tunnel without causing additional mechanical stress [40]. The micro- and nanoporous structures of hydrogels facilitate the diffusion of therapeutic agents, oxygen, and nutrients, ensuring sustained therapeutic effects. Pore sizes, typically 50–200 nm for drug delivery, can be precisely controlled through crosslinking techniques and polymer blending, allowing nutrient exchange while preventing fibrotic infiltration [41].

On the chemical front, hydrogels must demonstrate exceptional biocompatibility to minimize immune responses. Commonly used polymers, such as polyethylene glycol (PEG), hyaluronic acid, and alginate, are favored for their low immunogenicity [14]. Crosslinkers should not release toxic by-products during degradation, making bio-orthogonal or enzymatically degradable systems preferable over traditional covalent agents like glutaraldehyde [42]. The degradation rate of hydrogels, influenced by the type and density of crosslinks (e.g., ester or disulfide bonds), must align with the nerve healing timeline, typically requiring stability over weeks to months [43].

Functionalization enhances the therapeutic efficacy of hydrogels. The incorporation of bioactive agents, such as anti-inflammatory drugs (e.g., dexamethasone), neurotrophic factors (e.g., brain-derived neurotrophic factor, BDNF), or extracellular matrix components (e.g., collagen), allows targeted interventions [44]. Surface functionalization with cell-adhesive peptides, such as RGD sequences, promotes cellular interaction and tissue integration [45]. Smart hydrogels, responsive to environmental stimuli like pH, temperature, or mechanical stress, offer advanced therapeutic control. For instance, thermoresponsive hydrogels are based on poly(N-isopropylacrylamide) (PNIPAM) transition from sol to gel at body temperature, enabling localized drug delivery [46,47]. Similarly, pH-sensitive hydrogels release encapsulated agents in response to the acidic inflammatory microenvironment of CTS [48].

Crosslinking methods play a pivotal role in defining hydrogel properties. Chemical crosslinking, such as covalent bonding, provides high mechanical strength and stability [49,50]. Advanced techniques, including radical polymerization, click chemistry, and enzymatic reactions, offer precise control over network density, influencing elasticity and degradation rates [51]. Photo-crosslinking enables the spatial and temporal control of gelation, mimicking the heterogeneous stiffness of the carpal tunnel [52]. Ionic crosslinking, as seen in alginate-based hydrogels, allows reversible gelation but may require reinforcement with covalent bonds for long-term stability [53].

Physical crosslinking, relying on dynamic and reversible interactions like hydrogen bonding and van der Waals forces, imparts self-healing properties, valuable for repetitive motion in the wrist [54]. Thermo-gelling systems, such as PNIPAM-based hydrogels, enable minimally invasive delivery, transitioning from liquid to gel at physiological temperatures [55]. Hybrid crosslinking methods combining physical and chemical strategies, such as double-network hydrogels, provide robustness and adaptability, making them well suited for the repetitive loading environment of the carpal tunnel [56].

**Figure 2:**
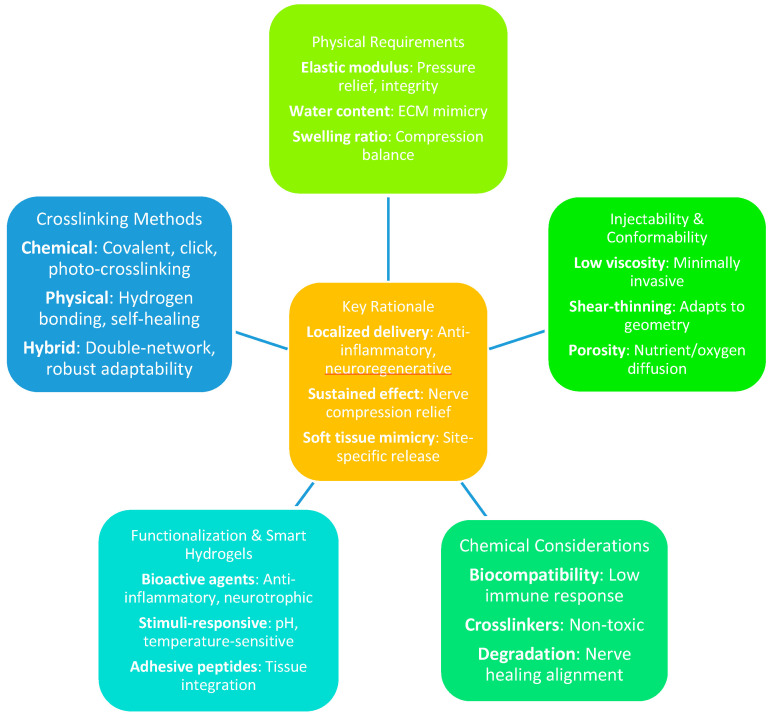
Overview of key design considerations for hydrogel-based CTS therapies.

The integration of physical and chemical properties in hydrogel design is essential for addressing the specific therapeutic requirements of CTS. Physically, hydrogels must provide cushioning and withstand mechanical stress, while chemically, they must enable controlled drug release, promote biodegradation, and support tissue regeneration. By optimizing crosslink density, polymer composition, and functionalization, hydrogels can address both the mechanical and biological challenges of CTS. This synergy opens pathways for innovative, effective therapies tailored to the complex demands of CTS. Future research should focus on refining these parameters through advanced modeling and experimental validation to fully realize the clinical potential of hydrogels (Figure 2).

## 3. Carpal Tunnel Syndrome: Pathophysiology and Treatment Overview

CTS arises from the pathological compression of the median nerve within the carpal tunnel, often precipitated by inflammation, edema, or hypertrophy of the synovial tissues surrounding the flexor tendons [57]. This compression compromises neural vascularization, creating ischemic conditions that lead to sensory and motor deficits in the hand and fingers [58]. The pathophysiology of CTS extends beyond mechanical entrapment, involving a complex interplay of inflammatory processes, myelin degradation, and progressive nerve fiber degeneration [59]. Inflammatory mediators such as tumor necrosis factor-alpha (TNF-α) and interleukin-1 beta (IL-1β) contribute to a pro-inflammatory environment, exacerbating tissue swelling and nerve damage while amplifying ischemic effects on the median nerve [60].

Ischemia further complicates CTS by restricting blood flow, which deprives nerve tissues of oxygen and nutrients, ultimately accelerating axonal damage [61]. This vascular compromise disrupts nerve signaling and renders nerve fibers more susceptible to degeneration [57]. Such interconnected mechanisms underscore the challenge of effectively treating CTS, as interventions must address both mechanical compression and the underlying inflammatory and ischemic factors contributing to nerve dysfunction [62].

Existing treatment paradigms predominantly target symptom relief and mechanical decompression [63]. First-line conservative therapies—such as nocturnal splinting, corticosteroid injections, and physical therapy—often provide only temporary symptom alleviation, leaving structural and inflammatory pathologies unresolved [64,65]. Although developments like ultrasound-guided corticosteroid injections and newer pharmacological agents have improved symptom management, they still fall short of delivering sustained efficacy without confronting the disorder’s core pathophysiological components [66].

Surgical approaches, including open and endoscopic carpal tunnel release, aim to decompress the median nerve definitively by severing the transverse carpal ligament [6]. While successful for many patients, these procedures carry risks such as postoperative scar formation, fibrosis, chronic pain, and extended recovery periods [67]. Moreover, outcomes can be unpredictable, with some patients experiencing the incomplete restoration of nerve function or symptom recurrence [68]. These limitations highlight the critical need for less invasive, more targeted therapies that can mitigate symptoms while promoting long-term nerve health.

The urgency of regenerative therapies for CTS is increasingly recognized. Effective interventions must not only ameliorate symptoms but also support the repair and regeneration of compromised nerve fibers and myelin [69]. This is especially challenging as nerve healing requires both structural and biochemical support. Innovative strategies such as hydrogels, capable of delivering localized anti-inflammatory and neuroregenerative compounds, present a promising avenue [28]. By offering controlled, site-specific drug release within the carpal tunnel, hydrogels may address inflammation and nerve damage more effectively than traditional conservative or surgical methods.

**Figure 3:**
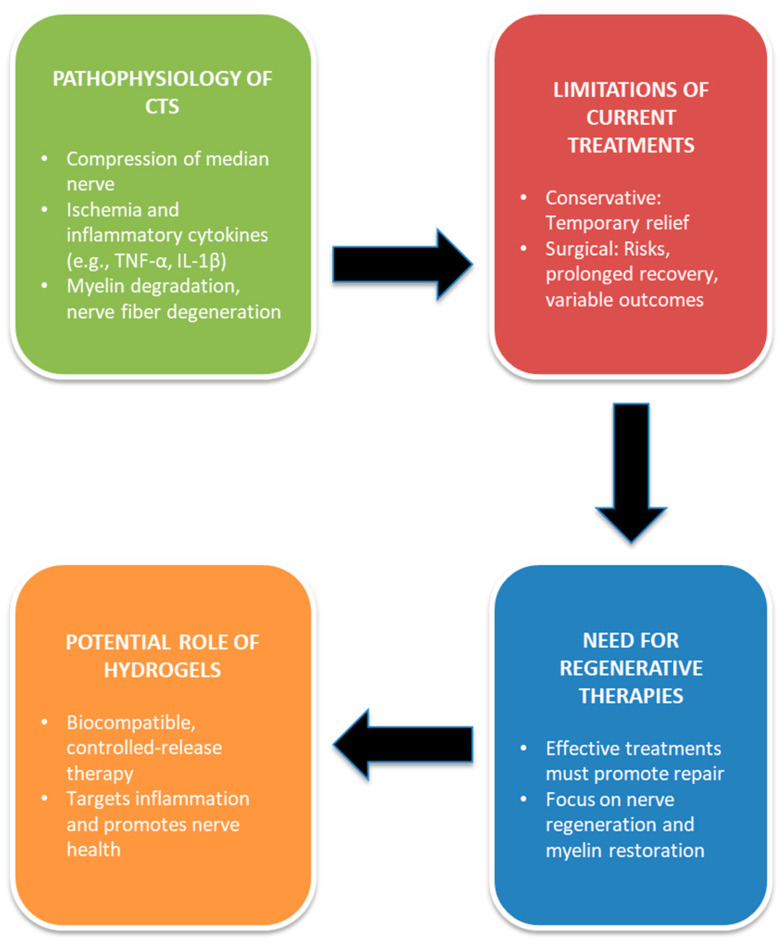
Pathophysiological challenges of CTS and the therapeutic potential of hydrogel.

Given the multifaceted nature of CTS pathology and the limitations of current treatments, there is strong rationale for exploring minimally invasive solutions that directly target the foundational causes of nerve compression and degeneration. Hydrogels, with their biocompatibility, controlled-release properties, and localized treatment potential, represent a novel therapeutic pathway. By creating a regenerative microenvironment and reducing the need for repeated procedures, hydrogels may fundamentally redefine CTS management (Figure 3).

## 4. Hydrogels in the Treatment of Carpal Tunnel Syndrome

Peripheral nerve disorders, including CTS, present complex management challenges, particularly when the restoration of both structural and functional aspects of the nerve is paramount [70]. Although direct research on hydrogel-based therapies for CTS remains limited, the extensive application of hydrogels in other peripheral nerve conditions offers valuable insights. Hydrogels possess unique attributes—such as biocompatibility, tunable mechanical properties, and the capacity to deliver therapeutic agents—that make them promising tools for targeting the critical pathophysiological features of CTS [71]. By drawing parallels from relevant nerve injury studies and aligning them with the specific pathophysiological mechanisms of CTS, a clearer understanding emerges of how hydrogels might be employed to advance CTS treatment.

### 4.1. Insights from Related Peripheral Nerve Conditions

Exploring hydrogel applications in other peripheral nerve conditions can provide valuable insights into their potential role in CTS therapy. These conditions often share similar pathophysiological features with CTS, including mechanical compression, inflammation, fibrosis, and nerve degeneration [58]. By examining how hydrogels are employed in these contexts, we can glean important information about their adaptability and utility in CTS treatment.

Peripheral nerve injuries, regardless of their anatomical location, typically involve the same core challenges: restoring nerve function, reducing inflammation, and preventing scar tissue formation [72]. Addressing these issues requires creating a supportive environment that promotes nerve repair while minimizing external obstacles, such as excessive mechanical strain or fibrotic encroachment [73]. Hydrogels, with their inherent adaptability, serve as a versatile scaffold for tackling these problems. Their biocompatibility, combined with tunable mechanical and chemical properties, makes them a powerful tool in peripheral nerve repair [23].

Insights from other peripheral nerve conditions highlight the potential of hydrogels as a multifunctional therapeutic platform. Their capacity to incorporate anti-inflammatory, anti-adhesion, and regenerative features makes them an appealing option for addressing the multifaceted pathophysiology of CTS, thereby paving the way for innovative, minimally invasive therapies [28].

### 4.2. Comparative Analysis of Carpal Tunnel Syndrome and Generalized Peripheral Nerve Injuries: Implications for Hydrogel Applications

Despite several shared features, CTS differs significantly from generalized peripheral nerve injuries in its anatomical, biomechanical, and pathological characteristics, necessitating tailored therapeutic strategies. In CTS, the median nerve is chronically compressed within the rigid, narrow carpal tunnel—a situation worsened by repetitive hand and wrist movements [74]. This unique environment fosters inflammation, ischemia, and fibrosis, culminating in impaired nerve gliding and progressive nerve dysfunction [58]. By contrast, generalized peripheral nerve injuries often occur in less confined regions, such as the limbs, and typically arise from acute trauma (e.g., transections, crush injuries, or stretching) that disrupt nerve continuity and require large-scale scaffolding for regeneration [72]. The chronic and localized nature of CTS sets it apart: sustained compression triggers inflammation and hypoxia, which perpetuate fibrosis and axonal degeneration [75].

To address these challenges, hydrogel therapies must be specifically designed for CTS. Unlike hydrogels developed for generalized nerve injuries, often intended to bridge extensive nerve gaps, CTS-focused hydrogels must be miniaturized to fit within the narrow carpal tunnel without exacerbating compression [71]. Hydrogels with viscoelastic properties can withstand repetitive stress and maintain their structural integrity, while anti-adhesion formulations can prevent fibrosis and support smooth nerve gliding [76]. Additionally, CTS-specific hydrogels must emphasize localized drug delivery to manage chronic inflammation and encourage nerve regeneration. Existing technologies, such as stimuli-responsive hydrogels used in spinal cord injuries or drug delivery systems in sciatic nerve injuries, can be adapted for CTS by refining their mechanical properties and release mechanisms to accommodate the carpal tunnel’s unique biomechanical constraints. For instance, biodegradable hydrogels can be tailored for sustained release of anti-inflammatory agents or growth factors (e.g., nerve growth factor) to promote axonal repair [77]. Incorporating patient-specific factors, such as diabetes or repetitive strain injuries, into hydrogel designs could further enhance therapeutic relevance.

Future research should prioritize creating CTS-specific animal models that mimic the cramped environment and repetitive stress characteristic of the carpal tunnel, enabling the precise evaluation of hydrogel efficacy [26]. Imaging and electrophysiological assessments can then measure improvements in nerve gliding, functional recovery, and inflammation reduction, while preclinical and clinical trials can validate hydrogels as adjuncts to surgical decompression. By aligning hydrogel design with the distinct requirements of CTS, these innovative materials may bridge the gap between broader peripheral nerve research and effective, minimally invasive CTS therapies. Such advancements could reshape the treatment paradigm, leading to better patient outcomes and alleviating the socioeconomic burden of CTS.

### 4.3. Linking the Pathophysiology of CTS to Hydrogel Applications

To fully appreciate how hydrogels may be utilized in the treatment of CTS, it is crucial to examine the specific pathophysiological mechanisms underlying the disorder. CTS arises from the chronic compression of the median nerve as it traverses the carpal tunnel [57]. Over time, this condition progresses through several inter-related processes, including mechanical compression, inflammation, perineural fibrosis, demyelination, and axonal degeneration. Each of these hallmark features presents a potential target for hydrogel-based interventions, offering new avenues for more effective CTS management [78].

#### 4.3.1. Mechanical Compression

The mechanical compression of the median nerve in CTS leads to localized ischemia and hypoxia, which disrupt normal nerve function [1]. Over time, repeated mechanical stress worsens nerve damage, resulting in demyelination and eventual axonal degeneration [79]. Hydrogels offer a novel strategy by creating a cushioning effect around the nerve, thereby reducing mechanical stress. In a recent study, Hsiao et al. employed perineural hydrogel injections to model entrapment neuropathy, providing insights that may be adapted for CTS treatment [26]. Building on this concept, anti-adhesion hydrogels could be engineered to prevent scar tissue formation and enhance nerve gliding.

Dong et al. developed an electrospun polycaprolactone–amnion nanofibrous membrane that successfully reduced adhesions and supported nerve repair in a rat model of sciatic nerve compression [80]. This membrane mimicked the extracellular matrix, minimized fibrosis, and promoted cell growth, underscoring its potential for treating conditions like CTS by preventing scar formation and enhancing nerve recovery in a minimally invasive manner. In another study, Li et al. formulated a double-network hydrogel to alleviate lumbar nerve root compression in a rat model [81]. By providing mechanical support and reducing nerve stress during dynamic compression, the hydrogel improved nerve function and lowered inflammation, as indicated by histological and electrophysiological assessments. These findings highlight the suitability of hydrogel-based strategies for compression-related neuropathies, including CTS.

The carpal tunnel poses unique mechanical challenges due to its confined space, repetitive motion, and susceptibility to elevated pressure from surrounding tissues [74]. These conditions give rise to inflammation, fibrosis, and reduced nerve gliding. Hydrogels can address these issues by delivering load-bearing support, minimizing friction, and preventing adhesions. Specifically, hydrogels with viscoelastic properties can withstand repetitive stress, maintaining structural integrity within the carpal tunnel’s dynamic environment [82]. Anti-adhesion hydrogels further reduce the formation of scar tissue, aiding in smooth nerve gliding and decreasing secondary compression [71]. Moreover, hydrogels engineered with microscale porosity can improve nutrient and oxygen diffusion, mitigating the hypoxic stress that exacerbates nerve damage [83]. Through these mechanical adaptations, hydrogels emerge as a promising strategy for alleviating the physical constraints of the carpal tunnel, thereby promoting nerve health and supporting long-term functional recovery.

#### 4.3.2. Inflammation

Inflammation is a key factor in the pathophysiology of CTS, often triggered by mechanical stress and ischemia. Prolonged inflammation activates fibroblasts, contributing to perineural scarring and adhesion formation, which worsen nerve compression [57]. Hydrogels can be engineered to deliver anti-inflammatory agents directly to the affected region, thus mitigating these processes. For instance, Zhijiang He and colleagues demonstrated that a hyaluronan–methylcellulose hydrogel modified with an anti-inflammatory peptide and brain-derived neurotrophic factor (BDNF) promoted nerve regeneration in a rat model of spinal cord injury [84]. Adapting this strategy for CTS could involve designing a hydrogel that releases anti-inflammatory compounds and BDNF specifically around the compressed median nerve within the carpal tunnel. Such an approach would need to be optimized to match the wrist’s anatomical constraints, providing a supportive scaffold for nerve healing without hindering nerve movement.

A similar concept has been explored by Javanmardi and colleagues, who developed a composite hydrogel system combining dexamethasone-loaded hyaluronic acid microparticles with a proanthocyanidin–gelatin matrix. This formulation effectively supported sciatic nerve regeneration in a rat model [85]. A dexamethasone-releasing hydrogel tailored for CTS could potentially reduce inflammation, support nerve repair, and improve mobility, offering a minimally invasive, patient-friendly alternative to surgery.

Kong et al. took another approach by creating an in situ-forming, curcumin-loaded hydrogel for treating chronic peripheral neuropathy in rats [86]. This hydrogel targeted inflammation through localized curcumin delivery, lowering pro-inflammatory cytokines and fostering a supportive environment for nerve recovery. Histological analysis confirmed a reduction in inflammation and improved nerve health, suggesting that a similar approach could be adapted for CTS by targeting inflammatory processes around the median nerve.

Additionally, Dong et al. developed an injectable, adaptable hydrogel capable of releasing hydrogen sulfide (H_2_S) in a controlled manner, modulating the neuroregenerative microenvironment in a rat model of sciatic nerve injury [87]. This approach reduced inflammation and oxidative stress, thereby promoting nerve repair. The adaptability and sustained release properties of such hydrogels underscore their potential for CTS management, as they can create a favorable environment for nerve regeneration in a confined space like the carpal tunnel.

#### 4.3.3. Perineural Fibrosis

Perineural fibrosis frequently complicates CTS, especially following surgical decompression. The resulting scar tissue restricts nerve mobility and can contribute to symptom recurrence [88]. Anti-adhesion hydrogels—such as those derived from CMC-PE—have proven effective in preventing perineural adhesions in animal models of sciatic nerve injury. By forming a physical barrier around the nerve, these hydrogels inhibit scar formation without disrupting the natural healing process [71]. Incorporating such hydrogels during carpal tunnel release surgery could potentially reduce recurrence rates and improve long-term outcomes.

In a related study, Zhan et al. developed a polydopamine nanoparticle-loaded hyaluronic acid methacryloyl hydrogel for photothermal therapy, specifically designed to prevent peripheral nerve adhesion in a rat sciatic nerve model [89]. The photothermal effect reduced perineural fibrosis and inflammation, thereby promoting nerve regeneration. This technique may hold particular promise for CTS by mitigating scar formation and supporting nerve healing.

#### 4.3.4. Demyelination and Axonal Damage

Advanced stages of CTS involve demyelination and axonal degeneration, contributing to both sensory and motor deficits [1]. Conductive hydrogels have shown particular promise in addressing these complications in peripheral nerve injury models. By providing a supportive matrix for axonal regrowth and remyelination, these hydrogels can accelerate the restoration of nerve function [90]. In the context of CTS, combining conductive hydrogels with electrical stimulation could further enhance the repair of demyelinated fibers and improve functional outcomes.

Wu et al. developed a functional self-assembling peptide nanofiber hydrogel to promote peripheral nerve regeneration. In a rat sciatic nerve injury model, the hydrogel created a favorable environment for axonal regrowth, resulting in better nerve function [91]. This suggests its potential for treating nerve injuries like CTS by supporting axonal repair and functional recovery. Similarly, Yang et al. fabricated self-assembling peptide hydrogels functionalized with laminin (LN) and BDNF mimetic epitopes, which enhanced peripheral nerve regeneration in a rat sciatic nerve model [92].

Yao et al. advanced this concept by developing a magnesium-encapsulated injectable hydrogel combined with a 3D-engineered polycaprolactone (PCL) conduit. In a rat sciatic nerve defect model, this composite promoted axonal regrowth and improved functional recovery [93]. Meanwhile, Liu et al. designed a nerve guidance conduit incorporating fibrin hydrogels loaded with Wnt5a, leading to significantly enhanced axonal regrowth and function in a rat sciatic nerve injury model [94].

Lastly, Qiu et al. introduced adhesive chitosan-based hybrid biohydrogels to repair peripheral nerve injuries, demonstrating a notable increase in axonal regeneration and improved structural and functional recovery in a rat sciatic nerve model [19]. By providing a supportive microenvironment for Schwann cell proliferation—vital to axonal repair—these hydrogels underscore their potential utility in treating CTS and other nerve injuries.

## 5. Conclusions

Hydrogels hold considerable promise as transformative therapeutic tools for addressing the complex, multifactorial pathology of CTS. By leveraging their biocompatibility, adaptability, and controlled-release properties, hydrogels can help overcome key challenges in CTS management, including mechanical nerve compression, chronic inflammation, perineural fibrosis, and axonal degeneration. Despite this potential, the use of hydrogels in CTS-specific contexts remains in its infancy, partly due to limited translational data. A major challenge lies in the shortage of CTS-specific animal models that accurately replicate the unique anatomical, biomechanical, and pathological features of median nerve compression. Consequently, current research often extrapolates findings from generalized peripheral nerve injury models that do not capture the distinct clinical and pathophysiological nuances of CTS.

To address these limitations, future research should prioritize the development and evaluation of hydrogels tailored to CTS. Such hydrogels could include localized drug delivery systems with sustained release profiles, anti-adhesion barriers to reduce scarring and fibrosis, and regenerative scaffolds to support nerve repair and functional recovery. Advanced hydrogel technologies—such as stimuli-responsive and biofunctionalized formulations—may further enhance therapeutic precision and effectiveness.

When adapting hydrogel-based treatments for CTS, the carpal tunnel’s narrow, confined space and elevated mechanical stress from repetitive hand movements must be taken into account. Unlike larger nerve areas, hydrogels for CTS must remain flexible and avoid excessive swelling to prevent additional compression of the median nerve. Strategies employed in models of larger nerve injuries, such as targeted drug delivery for the sciatic nerve, can be adapted by miniaturizing and refining mechanical properties to suit the carpal tunnel. Stimuli-responsive hydrogels used in spinal cord injury therapies can likewise be optimized for CTS by integrating viscoelastic materials that withstand repetitive wrist motion while providing localized therapeutic delivery.

Therapeutic hydrogels may be further modified to incorporate anti-inflammatory agents or growth factors that promote healing and nerve regeneration. CTS-specific animal models could then replicate the biomechanical constraints of the carpal tunnel, enabling rigorous assessments of hydrogel efficacy. Biodegradable hydrogels could minimize long-term complications, while imaging and electrophysiological testing could evaluate improvements in nerve gliding and functional recovery. Incorporating patient-specific factors, such as diabetes or repetitive strain, would enhance the clinical relevance of these approaches. Future preclinical and clinical studies might explore hydrogels as adjuncts to surgical decompression, potentially preventing postoperative adhesions and improving recovery, thereby facilitating the translation of hydrogel technologies into minimally invasive, effective CTS therapies that support long-term nerve health.

Incorporating hydrogel-based interventions alongside complementary treatments—such as neuroprotective drugs, physical rehabilitation, and surgical decompression—may create synergistic therapeutic approaches. Collaboration among biomedical researchers, clinicians, and regulatory bodies is essential to expedite the transition of hydrogel-based solutions from laboratory research to clinical practice. Establishing standardized manufacturing protocols, ensuring regulatory compliance, and conducting robust preclinical and clinical trials will be vital for advancing this field.

## Figures and Tables

**Table 1:** Types of hydrogels according to CTS-specific benefits.

Category	Description	Examples	CTS-Specific Benefits
Types of Hydrogels	- Synthetic: Precisely tunable properties [17] - Natural: Enhanced biocompatibility [18] - Hybrid: Combination of synthetic and natural polymers [19]	Polyacrylamide (PAA), polyvinyl alcohol (PVA), polyethylene glycol (PEG), alginate, chitosan, collagen, synthetic/natural composites	Tunable strength and degradation suited for carpal tunnel constraints; natural cell affinity promotes tissue integration and regeneration
Key Properties	- Hydrophilicity: Retains water, mimics biological tissue for reduced immunogenicity and toxicity [20] - Controlled Drug Release: Adjusted through polymer composition and crosslinking [21] - Biodegradability: Degrades over time in vivo, reducing need for removal [22]		Hydrated state supports compatibility with soft tissue, minimizing inflammatory response in carpal tunnel; enables targeted, sustained release of anti-inflammatory or neuroregenerative agents; gradual degradation can provide prolonged support and reduce long-term interventions
Applications in Medicine	- Drug Delivery: Localized and controlled release systems [23] - Tissue Engineering: Supports cellular growth, wound healing, and regenerative treatments [24] - Soft Tissue Repair: Conforms to complex anatomical structures like joints or nerves [25]		Allows direct delivery of therapeutics to carpal tunnel area for CTS treatment; promotes regeneration and repair of nerve tissues affected by CTS; surface conformability allows close fit within carpal tunnel for effective treatment
Specific CTS Benefits [26]	- Localized Delivery: Direct application to carpal tunnel allows site-specific, minimally invasive therapy - Sustained Efficacy: Controlled release ensures prolonged therapeutic effect, essential for managing chronic CTS symptoms		Reduces need for systemic treatment, lowering side effects and improving patient outcomes; minimizes recurrence of symptoms, potentially delaying or preventing need for surgical intervention

## Data Availability

No new data were created or analyzed in this study. Data sharing is not applicable to this article.
